# Effects of Dietary Pomegranate Peel on Antioxidant Gene Expression and DJ-1 Protein Abundance in Ram Testes

**DOI:** 10.22074/IJFS.2021.141725.1052

**Published:** 2021-10-16

**Authors:** Mahdieh Nikfarjam, Leila Rashki Ghaleno, Abdolhossein Shahverdi, Shahab Mirshahvaladi, Seyed Mehdi Ghoreishi, AliReza Alizadeh

**Affiliations:** 1. Department of Embryology, Reproductive Biomedicine Research Center, Royan Institute for Reproductive Biomedicine, ACECR, Tehran, Iran; 2. Department of Cellular and Molecular Biology, Faculty of Basic Sciences and Advanced Technologies in Biology, University of Science and Culture, Tehran, Iran; 3. Reproductive Epidemiology Research Center, Royan Institute for Reproductive Biomedicine, ACECR, Tehran, Iran; 4. Department of Stem Cells and Developmental Biology, Cell Science Research Center, Royan Institute for Stem Cell Biology and Technology, ACECR, Tehran, Iran; 5. Department of Clinical Medicine, Faculty of Medicine and Health Sciences, Macquarie University, Sydney, Australia; 6. Department of Animal Science, School of Agriculture, Shiraz University, Shiraz, Iran

**Keywords:** Antioxidant Genes, Pomegranate, Ram Testes

## Abstract

**Background:**

Pomegranate is an ancient fruit containing Punicalagin, which has known as an effective antioxidant. Pomegranate peel was recognized as a phenol and tannin source, and pomegranate seed contains unique fatty acid (Punicic acid). Limited information exists about the influences of pomegranate peel and seed on antioxidant enzymes and proteins in the male reproduction system. This study was performed to determine the pomegranate peel and seed effects on the expression of antioxidant genes and DJ-1 protein in ram’s testis.

**Materials and Methods:**

In this experimental study, twenty-one mature Iranian rams were randomly divided into three groups (n=7 in each group), and fed experimental diets consisted of a control diet (C), a diet containing dry pomegranate seed pulp (S), and a diet containing pomegranate peel (P) for 80 days. All rams were offered isoenergetic and isonitrogenous rations. Testicular tissue samples were collected, and expression of *Gpx1, Gpx4, Prdx4, Prdx5,* and *Sod2* genes was quantified by real-time polymerase chain reaction (RT-PCR). In addition, western blotting was used to evaluate DJ-1 expression at the protein level.

**Results:**

*Gpx1* and *Sod2* mRNA levels in the peel group were significantly (P<0.05) higher than control. *Prdx5* mRNA level was increased (P<0.05) in the seeds group than in the control group. *Gpx4* and *Prdx4* expression were statistically not affected significantly by the experimental diet. Data analysis showed a significant (P<0.05) increase (1.5-fold) in the expression level of DJ-1 in peel groups than in control.

**Conclusion:**

The expression of antioxidant genes and DJ-1 protein in ram testes are more influenced by pomegranate peel than seed.

## Introduction

Infertility is a critical problem in couples' lives who are demanding a child. At present, near 7% of couples in the world are infertile, and half of these cases are related to male factors (1). In recent decades, extensive research and numerous publications have demonstrated pathological levels of reactive oxygen species (ROS) and oxidative stress (OS) following the weak potential of defense in the seminal plasma leading to spermatozoa damage and male infertility (2). In addition to the harmful effects of OS on sperm classical parameters like motility, morphology, and DNA, a high level of ROS can impair spermatogenesis and sperm maturation by the alternation of *H19* gene methylation (3).

Antioxidants enzymes are the only mechanism that protects sperm against the damaging effects of OS. Superoxide dismutase (SOD), glutathione (GSH), and catalase (CAT) are well-known enzymatic antioxidants in the seminal plasma and sperm, encoded by *NRF2* (4), *SOD, CAT,* glutathione S-transferase (*GST*), and glutathione peroxidase (*GPX*) genes. Alteration in these genes may lead to male infertility, and it seems that the improved activity of these genes may improve male fertility (5).

Antioxidant therapy and oral antioxidants are the most frequent suggestions to control male infertility induced by OS. In an ideal world, oral antioxidants in high concentration could improve spermatogenesis in the reproductive tract. Besides, they should increase the capacity of the seminal plasma clearance and leads to the reduction of ROS level in semen [reviewed by (6)]. In addition to synthetic antioxidants, herbal antioxidants such as Saffron (*Crocus sativus*) and Pomegranate (*Punica granatum*) positively affected sperm parameters such as motility and morphology (7, 8). Pomegranate from the Punicacea family is an ancient and aboriginal fruit of Iran known as a great antioxidant and used to treat several diseases such as dysentery or respiratory pathologies. Pomegranate has numerous polyphenols, including anthocyanins, minor flavonoids, and punicalagin in the peel, seed, and juice (9). The limited evidence available regarding the role of each component of this fruit (peel or seed) and its possible mechanisms related to successful therapy for male infertility. Some reports have shown that daily consumption of pomegranate juice increases the number of spermatogonia cells, sperm motility and decreases sperm lipid peroxidation in male rats (10). Moreover, using pomegranate juice could increase the expression level of *Sod, Gpx, Gst,* and *Gsh* antioxidant enzymes in male rat testis (11). On the other hand, dietary supplementation of pomegranate seed for cloned male goats could decrease OS and improve sperm motility, viability, and following sperm cryopreservation in this species (12).

Nevertheless, a higher proportion of presented data in papers is based on pomegranate juice consumption in animal models; peel or seed's effect and antioxidant effects on testis are still unclear. Furthermore, to our best knowledge, whether the inclusion of pomegranate peel (the source of polyphenols and tannins) or pomegranate seed (the source of unique fatty acid [FA]; Punicic acid) in the diet affects gene expression in testes has not been addressed.

With this background, the present research was performed to study the antioxidant effects of pomegranate peel, and seed on the expression of *Gpx1, Gpx4, Prdx4, Prdx5,* and *Sod2* genes and DJ-1 protein in testis following feeding rams with a daily diet contained pomegranate peel or pomegranate seed.

## Materials and Methods

### Preparation of pomegranate

In this experimental study, the pomegranate peels were freshly provided by the Sunich (Saveh, Iran). Pomegranate peels silage was prepared by mixing 95% pomegranate peels, 3% wheat straw, and 2% urea. Dried pomegranate seeds were bought from a local factory (Narni, Neyriz, Iran). Total tannins and phenolic compounds were measured by methods which are defined by Makkar (13) at Animal Science Research Institute of Iran, Karaj, Iran. The total phenol content and total tannin content of pomegranate peel were 3.09 and 1.81 percent, respectively. FA profiles in peels and seeds were determined at the Institute of Medicinal Plants, ACECR, Karaj, Iran by Gas Chromatography/Mass Spectrometry (GC/ MS) (Agilent GC 6890 system, Agilent Technologies Co., Hewlett Packard, Wilmington, DE, USA) (Figes.S1, S2, Table S1, See Supplementary Online Information at www.ijfs.ir).

### Animals and experimental design

Following approval of study protocol by the Ethics committee of Royan Institute (IR.ACECR.ROYAN. REC.1395.143); twenty-one Iranian fat-tailed rams (8 months of age; 27.03 ± 3.5 kg body weight) were housed in individual pens under a protective condition in the Animal Research Station, College of Agriculture, Shiraz University, Shiraz, Iran.

Rams were randomly divided into three groups (n=7 in each group); group I: control (basal diet without supplements), group II: pomegranate peel group (a diet containing 27% pomegranate peels silage), group III: pomegranate seed group (a diet containing 31% pomegranate seeds). All rations were isoenergetic and isonitrogenous. Animals received the diet as a total mixed ration (TMR) (according to National Research Council requirements of sheep and goats [NRC, 2007]), twice daily at 08:00 and 17:00 hours for 80 days (Table S2, See Supplementary Online Information at www.ijfs.ir). The first twenty days of the experiment was the adaptation period.

### Sample collection, RNA isolation, and real-time quantitative reverse transcription polymerase chain reaction

At the end of the experimental period, all rams were sacrificed. Testis was collected, snap-frozen in liquid nitrogen (196°C), and stored at - 80°C till further processing. Total RNA was isolated from testis tissues using TRIzol reagent (Invitrogen, USA) and dissolved in RNase-free water. The quantity and quality of extracted RNA were checked by a NanoDrop spectrophotometer (Thermo Fisher Scientific Inc., USA) and gel electrophoresis. Only samples with the A260/A280 ratio between 1.8 and 2 were used in this study. Two micrograms of total RNA from each sample were reverse transcribed into complementary DNA (cDNA) with random Hexamer primers using primeScriptTM1st strand cDNA synthesis kit (Takara, USA). The cDNA synthesized was kept at -20°C until needed. The transcript of selected genes was detected by real time-PCR step one plus Applied BioSystems and using SYBR Green qPCR Master Mix (Takara, USA). The optimal conditions of each reaction were as follows: pre- denaturation at 95°C for 10 minutes, denaturation at 95°C for 10 seconds (40 cycles), annealing at 60°C for 20 seconds, and extension at 72°C for the 20 seconds. Fold changes of expression were calculated using the 2^-∆∆ct^ method. Relative expression levels of target genes were normalized by *GAPDH* as the housekeeping gene (Table 1).

**Table 1:** Sequences of primers for the real-time polymerase chain reaction experiments


Gene	Primer sequence (5'-3')	Size (bp)

*Sod*	F: CTGCAAGGAACAACAGGTCT	190
	R: TTGGTGTACTTGGTGTAAGGC	
*Gpx1*	F: GGACTACACCCAGATGAATGACC	107
	R: CGTTCTTGGCGTTTTCCTGATG	
*Gpx4*	F: CGCAATGAGGCAAGACTGACG	131
	R: CGCATTACTCCCTGGCTCCTG	
*Prdx4*	F: AAGGACTATGGCGTATATCTGGAA	182
	R: GGGCAGACTTCTCCGTGTTT	
*Prdx5*	F: GGGAAGGAGACAGATTTGTTAC	114
	R: CACATTCAGGGATTTGACGAT	
*Gapdh*	F: GGAGAAACCTGCCAAGTATG	126
	R: TGAGTGTCGCTGTTGAAGTC	


### Evaluation of DJ-1 protein level

Protein was extracted from frozen testis tissue samples using the TRIzol extraction method. Total protein concentration was measured using the Bradford reagent with human serum albumin (HSA) as the standard protein. An equal amount (30 μg) of total protein from each sample was separated on 12% sodium dodecyl sulphate-polyacrylamide gel electrophoresis (SDS-PAGE) at 110 v for 3 hours and were then transferred onto PVDF membranes (Bio-Rad, USA) at 12 v for 16 hours. The membranes were blocked with 2% non-fat dry milk in TBST solution (20 mM Tris-HCL pH=7.4, 15 mM NaCl, and 0.1 TWEEN-20) for 1 hour at room temperature under agitation. The blots were incubated with primary antibody (Anti-PARK7/DJ1, 1:10000, Abcam, USA) and Goat anti-Mouse IgG (H+L) Poly-HRP Secondary Antibody, HRP (1:60000, Thermofisher, USA) for 1h at room temperature.

Protein visualization was carried out using the enhanced chemiluminescence (ECL) detection system (Amersham, ECL Healthcare Life Sciences, Little Chalfont, UK). The intensities of protein bands on the scanned X-ray films were quantified using the ImageJ software version 1.50i (US National Institutes of Health, Bethesda, USA). The changes in the DJ-1 level were normalized against β-actin as a housekeeping protein.

### Statistical analysis

Data were expressed as mean ± SEM. The normal distribution of data was confirmed by Kolmogorov-Smirnov’s test. Various parameters were compared to each other using One-way ANOVA. Multiple comparisons were performed using Post-hoc: Tukey range’s test. Statistical analysis was performed with the SPSS 20 software for Windows (SPSS Inc., Chicago, IL, USA). P<0.05 was considered statistically significant. The authors confirm that the data supporting the findings of this study are available within the article.

## Results

### Gene expression level of *Sod2, Gpx1, Gpx4, Prdx4*, and *Prdx5*

According to statistical analysis, the pomegranate peel group showed a significant increase in the expression level of *Sod2* (1.25 ± 0.06, P=0.01) and *Gpx1* (1.21 ± 0.06, P=0.02) in testes compared to the control group (Figes.1, 2). Contrary to *Sod2* and *Gpx1, Prdx5* level was significantly higher in the fed group with seed than the control group (1.17 ± 0.06, P=0.02, Fig .3). Nonetheless, mRNA abundance of *Gpx4* and *Prdx4* were not significantly affected by pomegranate peel or pomegranate seed inclusion in the diet compared to the control group (Figes.S3, S4, See Supplementary Online Information at www.ijfs.ir). Moreover, the comparison of pomegranate peel and seed results did not show any notable difference between these two groups.

**Fig 1:**
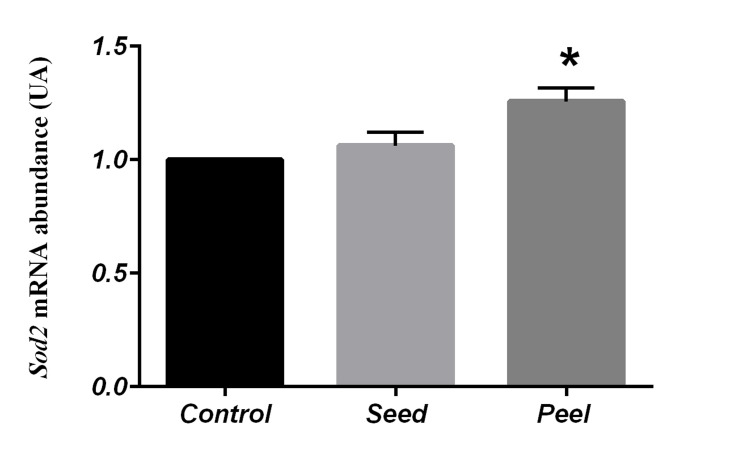
Comparison of Sod2 gene expression in control and fed groups with pomegranate seed and pomegranate peel using One Way-ANOVA (Posthoc test: Tukey’s range test). Data represented as mean values ± SEM. *; Indicates significant difference among the evaluated groups (P<0.05).

**Fig 2:**
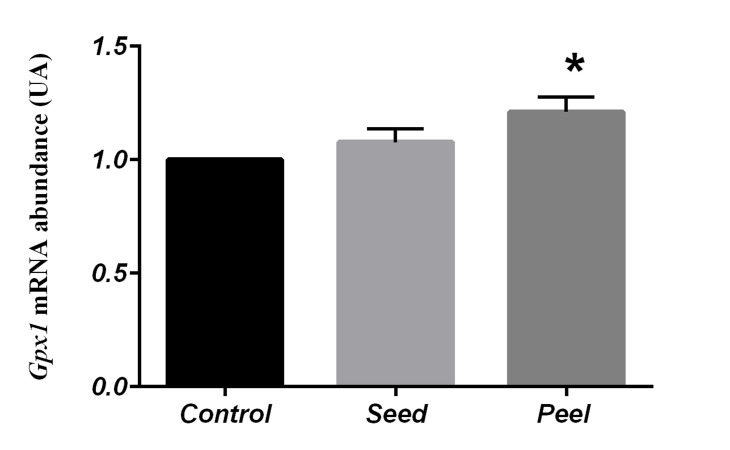
Comparison of Gpx1 gene expression in control and fed groups with pomegranate seed and pomegranate peel using One Way-ANOVA (Posthoc test: Tukey’s range test). Data represented as mean values ± SEM. *; Indicates significant difference among the evaluated groups (P<0.05).

**Fig 3:**
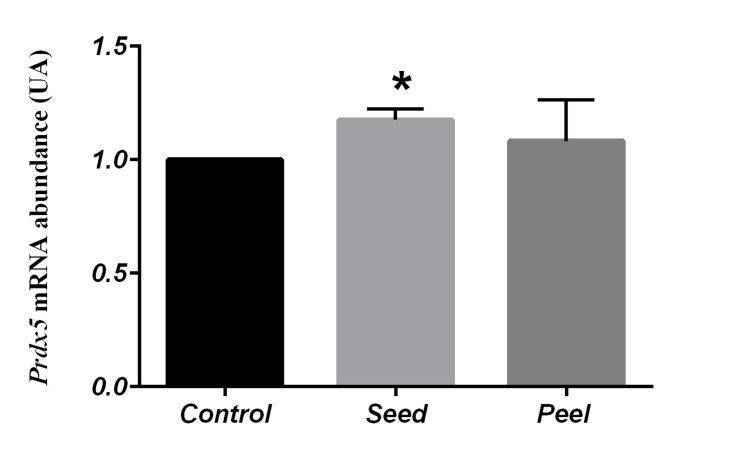
Comparison of *Prdx5* gene expression in control and fed groups with pomegranate seed and pomegranate peel using One Way-ANOVA (Post-hoc test: Tukey’s range test). Data represented as mean values ± SEM. *; Indicates significant difference among the evaluated groups (P<0.05).

### Level of DJ-1 protein

DJ-1 protein was elevated by pomegranate peel following the western blotting procedure (1.52 ± 0.22, P=0.04). However, the protein levels were unaltered by pomegranate seed (Figes.4, 5).

**Fig 4:**
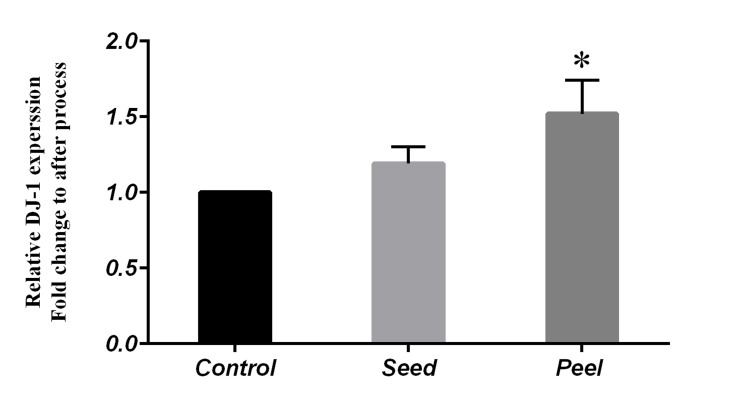
Comparison of relative DJ-1 expression in control and fed groups with pomegranate seed and pomegranate peel using One Way-ANOVA (Posthoc test: Tukey’s range test). Data represented as mean values ± SEM.*; Indicates significant difference among the evaluated groups (P<0.05).

**Fig 5:**
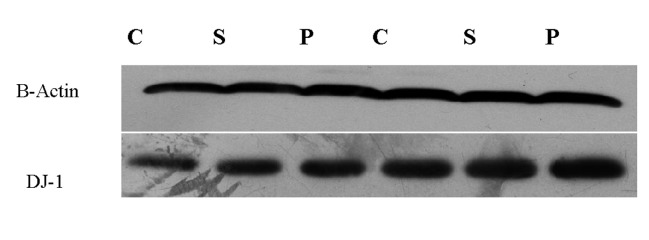
Western blot analysis of DJ-1 protein in control (C) and fed group with pomegranate seed (S) and peel (P).

## Discussion

This study provides compelling evidence on the effective use of pomegranate peel on the relative expression of various antioxidant genes in rams' testes. OS is one of the critical factors of male infertility that can disrupt testicular function and affect spermatogenesis. Antioxidants are the first line of protection in front of this unfavorable incident that can save testis and sperm against excessive ROS damage. Nowadays, consuming natural antioxidants, especially pomegranate products, as an aborigine group in the Middle East is one of the researchers’ interests in improving fertility in males. To better understand the pomegranate antioxidant mechanism on the testis, in the present study, pomegranate seed and peel effects were separately determined on antioxidant gene expression in Iranian fat-tailed rams testis, which fed pomegranate byproducts.

Although the expression of *Sod2, Gpx1, PrdX5* antioxidant genes, and DJ-1 protein had affected by adding seed and peel of pomegranate to the regular feeding plan of rams, the effect of peel was more notable about these genes’ expressions. Pomegranate has a high amount of polyphenol component of ellagitannins, especially punicalagin, as the most abundant soluble component of the peel with more than 50 percent potent antioxidant activity in pomegranate juice (14). As a putative mechanism, it was suggested that this polyphenol might increase the expression of Sod and Gpx antioxidant genes in testis, which has been reported in other species. Similarly, punicalagin and peel extract could reduce OS damage because of enhancement of the enzymatic capacity of SOD and GSH and decrease of lipid peroxidation in mouse (9) and rat testis (15), respectively. In this regard, Kang et al. showed the pomegranate powder through antioxidant inhibitory effects on melanin synthesis and tyrosinase activity, and the increase in Gpx1 may lead to prevention of melanogenesis in B16F10 melanoma cells via inactivation of the p38 signaling pathway (16).

In agreement with the mRNA behavior of *Sod* and *Gpx*, pomegranate peel significantly increased DJ-1 protein 1.5-fold compared to control. DJ-1 can protect cells and tissues against OS by enhancing the expression of several antioxidants such as SOD. In addition, this protein stabilizes Nuclear factor erythroid-2 related factor 2 (NRF2), a leading regulator for antioxidant proteins and detoxifying enzymes (17). Interestingly, pomegranate peel effectively increases DJ-1 protein level and antioxidant capacity in testis, which has not been reported before in previous studies. Therefore, the potential functions of peel were confirmed by western blot analysis and it could be proposed as a possible mechanism for the treatment of male infertility induced by OS and deficiency of DJ-1 function.

While this study revealed a high antioxidant potential for pomegranate peel according to a higher level of *Sod, Gpx,* and DJ-1, the seed had a more significant impact on *Prdx5* expression. *Prdx* family has a vital role in the safekeeping of cells against OS, especially hydrogen peroxide, and *Prdx4* and *Prdx5* have been found in spermatogonia (18). However, since there is no study regarding the effects of pomegranate’s by-products on *Prdx* family, it is not clear how pomegranate seed can increase the expression level of *Prdx5*. The unique profiles of FAs and the presence of Punicic acid in pomegranate seed may be a reason for such response in testis which warrants further studies.

## Conclusion

Even though both seed and peel of pomegranate are noteworthy in the stimulation of antioxidant capacity in testis, peel showed a higher impact on antioxidant genes such as *Sod2* and *Gpx1* as well as DJ-1 protein while seed only affected *Prdx5*. These findings will shed light and pave the way to acknowledging the importance of pomegranate by-products, especially pomegranate peel, as a natural antioxidant in the male antioxidant system. Besides clinical relevance, such research may result in considerable improvement in male infertility treatments.

## Supplementary PDF


