# Supplementary material for: Effects of Dietary Pomegranate Peel on Antioxidant Gene Expression and DJ-1 Protein Abundance in Ram Testes
Source: Int J Fertil Steril. 2021 Oct 16;15(4):258–62. doi: 10.22074/IJFS.2021.141725.1052 (PMC8530220; doi:10.22074/IJFS.2021.141725.1052)
Supplement: Supplementary file 1 [file Int-J-Fertil-Steril-15-258-s01.pdf]

Int J Fertil Steril
International Journal of Fertility & Sterility
ISSN:2008-076X eISSN:2008-0778

Effects of Dietary Pomegranate Peel on Antioxidant Gene Expression and DJ-1 Protein Abundance in Ram Testes

Mahdieh Nikfarjam, M.Sc.[1, 2#], Leila Rashki Ghaleno, M.Sc.[1#], Abdolhossein Shahverdi, Ph.D.[1, 3], Shahab Mirshahvaladi, M.Sc.[4, 5], Seyed Mehdi Ghoreishi, Ph.D.[6*], AliReza Alizadeh, Ph.D.[1*]

1. Department of Embryology, Reproductive Biomedicine Research Center, Royan Institute for Reproductive Biomedicine, ACECR, Tehran, Iran
2. Department of Cellular and Molecular Biology, Faculty of Basic Sciences and Advanced Technologies in Biology, University of Science and Culture, Tehran, Iran
3. Reproductive Epidemiology Research Center, Royan Institute for Reproductive Biomedicine, ACECR, Tehran, Iran
4. Department of Stem Cells and Developmental Biology, Cell Science Research Center, Royan Institute for Stem Cell Biology and Technology, ACECR, Tehran, Iran
5. Department of Clinical Medicine, Faculty of Medicine and Health Sciences, Macquarie University, Sydney, Australia
6. Department of Animal Science, School of Agriculture, Shiraz University, Shiraz, Iran

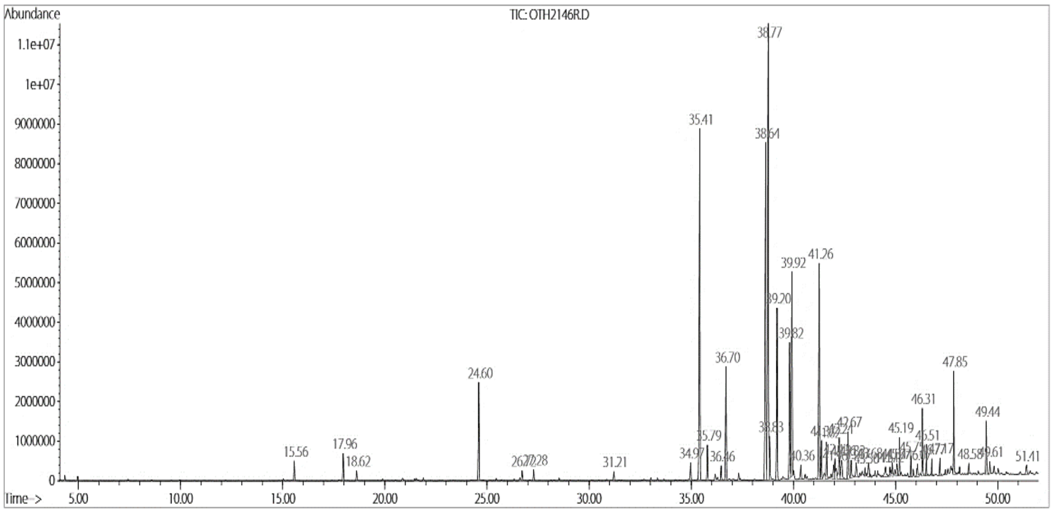

Fig.S1: Fatty acid chromatogram of pomegranate peel sample.

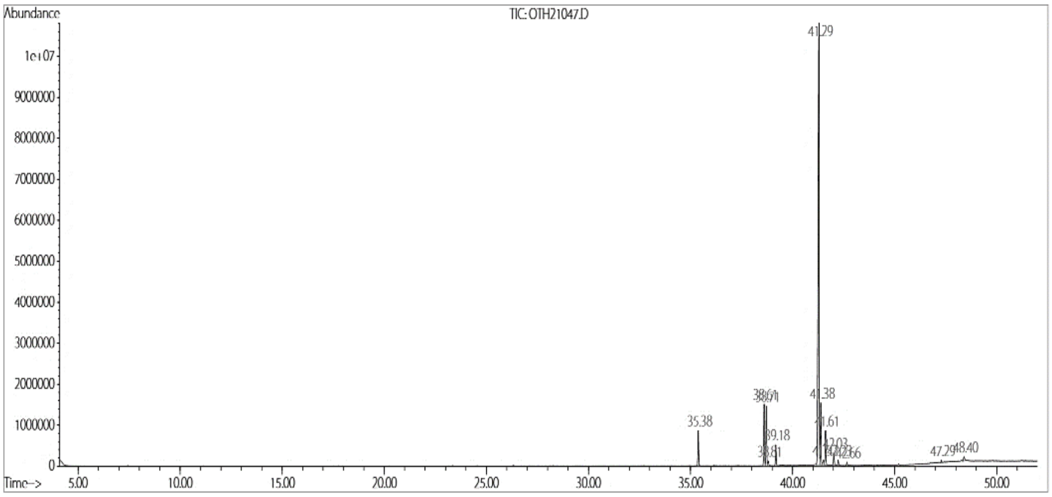

Fig.S2: Fatty acid chromatogram of pomegranate seed sample.

Received: 29/November/2020, Accepted: 9/May/2021
*Correspondening Addresses:
P.O.Box: 71441-65186, Department of Animal Science, School of Agriculture, Shiraz University, Shiraz, Iran
P.O.Box: 16635-148, Department of Embryology, Reproductive Biomedicine Research Center, Royan Institute for Reproductive Biomedicine, ACECR, Tehran, Iran
Emails: smghoreishi@shirazu.ac.ir, ar.alizadehmasouleh@royan-rc.ac.ir
#The first two authors equally contributed to this work.

Int J Fertil Steril
International Journal of Fertility & Sterility
ISSN:2008-076X eISSN:2008-0778

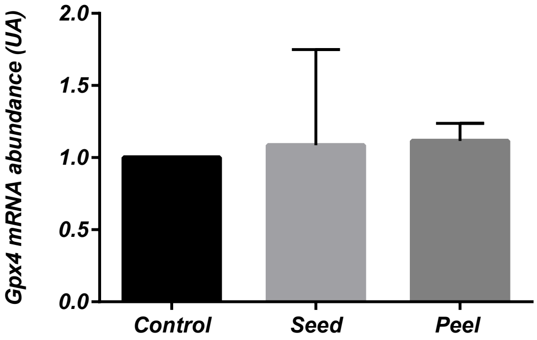

Fig.S3: Comparison of *Gpx4* gene expression in control and fed groups with pomegranate seed and pomegranate peel using One Way-ANOVA (Post-hoc test: Tukey's range test). Data represented as mean values ± SEM.

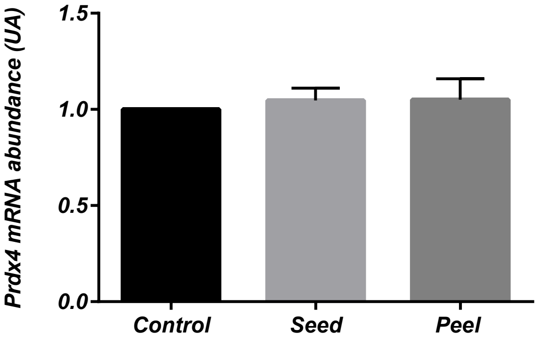

Fig.S4: Comparison of *Prdx4* gene expression in control and fed groups with pomegranate seed and pomegranate peel using One Way-ANOVA (Post-hoc test: Tukey's range test). Data represented as mean values ± SEM.

Table S1: Fatty acid profiles of peel and seed samples (% of total fatty acids)

Fatty acids	Peel	Seed
C8:0	0.54	ND
C14:0	0.27	ND
C16:0	16	3.61
C16:1	0.61	ND
C18:0	5.91	2.28
C18:1	23.8	6.90
C18:2	24	6.63
C18:3	ND	6.53
C18:3 (punicic acid)	ND	65
C20:0	3.12	0.63
C22:0	0.80	ND
C24:0	0.30	ND
Other fatty acids	31.84	8.48

ND; Not detectable.

Table S2: Ingredients (g/kg of dietary dry matter) and chemical composition of the experimental diets

Ingredients	Experimental diets		
	Control	Peel	Seed
Alfalfa hay	320	120	210
Corn grain	600	540	410
Wheat bran	70	54	56
Dried pomegranate seed	-	-	315
Pomegranate peel silage	-	275	-
Minerals and vitamins mix	10	11	9
Chemical composition (%)			
Dry matter	89	64	93
Crude protein	12	12	12
Neutral detergent fiber	38	45	46
Ash	6	5	5.5
Growth energy (Mcal/kg)	2.67	2.60	2.68
Calcium	0.63	0.46	0.47
Phosphorous	0.52	0.36	0.41
